# Association among pterygium, cataracts, and cumulative ocular ultraviolet exposure: A cross-sectional study in Han people in China and Taiwan

**DOI:** 10.1371/journal.pone.0253093

**Published:** 2021-06-15

**Authors:** Natsuko Hatsusaka, Naoki Yamamoto, Hisanori Miyashita, Eri Shibuya, Norihiro Mita, Mai Yamazaki, Teppei Shibata, Hidetoshi Ishida, Yuki Ukai, Eri Kubo, Hong-Ming Cheng, Hiroshi Sasaki

**Affiliations:** 1 Department of Ophthalmology, Kanazawa Medical University, Uchinada, Ishikawa, Japan; 2 Division of Vision Research for Environmental Health, Project Research Center, Medical Research Institute, Kanazawa Medical University, Uchinada, Ishikawa, Japan; 3 Department of Optometry, Asia University, Taichung, Taiwan; National Eye Institute, UNITED STATES

## Abstract

**Purpose:**

Pterygium is an ocular surface disorder mainly caused by ultraviolet (UV) light exposure. This study explored the relationships between six cataract types with pterygium and UV exposure.

**Methods:**

We have previously studied cataracts in residents of three regions in China and Taiwan with different UV intensities. From that study, we identified 1,547 subjects with information on the presence or absence of pterygium. Pterygium severity was graded by corneal progress rate. Cataracts were graded by classification systems as three main types (cortical, nuclear, posterior subcapsular) and three subtypes (retrodots, waterclefts, fiber folds) with high prevalence in middle-aged and elderly people. We calculated the cumulative ocular UV exposure (COUV) based on subject data and National Aeronautics and Space Administration data on UV intensities and used logistic regression to calculate odds ratios for the associations of COUV, cataract, and pterygium.

**Results:**

We found an overall pterygium prevalence of 23.3%, with significant variation among the three regions. Four cataract types (cortical, nuclear, posterior subcapsular, and retrodots) were significantly associated with the presence of pterygium.

**Conclusions:**

There was a significant association between COUV and pterygium, indicating that COUV is associated with the risk of pterygium development and that pterygium is useful as an index of UV exposure. Furthermore, the type of cataract in eyes with pterygium may indicate the level of UV exposure.

## Introduction

The ocular surface is exposed to various external factors, including three types of ultraviolet (UV) radiation produced by sunlight (UV-A, UV-B, and UV-C). UV-C light from the sun does not reach the surface of the Earth and accordingly not the human eye. Around 40% of UV-B is absorbed by the cornea on the ocular surface. UV-B is cytotoxic, and its absorption can result in cell death or abnormal cells by repetitive DNA damage and repair [1, 2]. The remaining 60% of UV-B and UV-A is mostly absorbed by the lens. UV-A is also known to cause oxidative damage to cell membrane lipids, proteins, and DNA [3].

UV-related eye diseases occur in the ocular parts of the keratoconjunctiva (cornea, conjunctiva) and lens. Eye diseases related to acute UV exposure include UV keratitis and snow blindness. Chronic UV exposure can lead to disorders such as cataract [4, 5], pterygium [6–8], and climatic droplet keratopathy [9–11]. A possible association of age-related macular degeneration with chronic UV-A exposure and light in the blue spectrum of solar radiation has also been suggested [12]. We previously reported the association between five types of cataract (three main and two subtypes) and UV exposure in the right eyes of Han people living in three regions of China and Taiwan (Sanya, Taiyuan, and Taichung), finding relationships between cumulative ocular UV exposure (COUV) and the prevalence of nuclear (NUC), cortical (COR), or posterior subcapsular cataracts (PSC) [13]. Many large-scale epidemiological studies have shown the relationship between ocular UV exposure and COR [5, 9, 14–17]. However, results for PSC have been mixed [18–20], and other studies reported that NUC is not related to UV exposure [9, 20]. However, there has been increasing evidence in recent years for the association of solar UV radiation with NUC [21].

Pterygium is an ocular disease in which subconjunctival tissues abnormally proliferate and wing-shaped fibrovascular tissues invade the corneal surface. The disease has been reported to be associated with UV radiation [22–27] and external stimuli [28–31]; recently, pathophysiological and genetic aspects have also been demonstrated [27, 32–37]. Few studies have examined the relationship between pterygium and cataract [38–40], and we are not aware of any that used UV radiation-related pterygium as an indicator to clarify the risk of UV exposure by cataract type.

The Han people are the largest ethnic group of China, Taiwan, and Singapore [41]. Studies have shown a higher risk of pterygium among the Han than among geographically similar ethnic groups such as the Uygur [42] and Mongols [43], while one study reported no significant difference in prevalence between the Manchu and Han people in China [44].

In the current study, we examined the relationship between pterygium and COUV in three geographical regions with different UV intensities, and between pterygium and cataract type in Han people.

## Materials and methods

### Subjects

**Fig 1:**
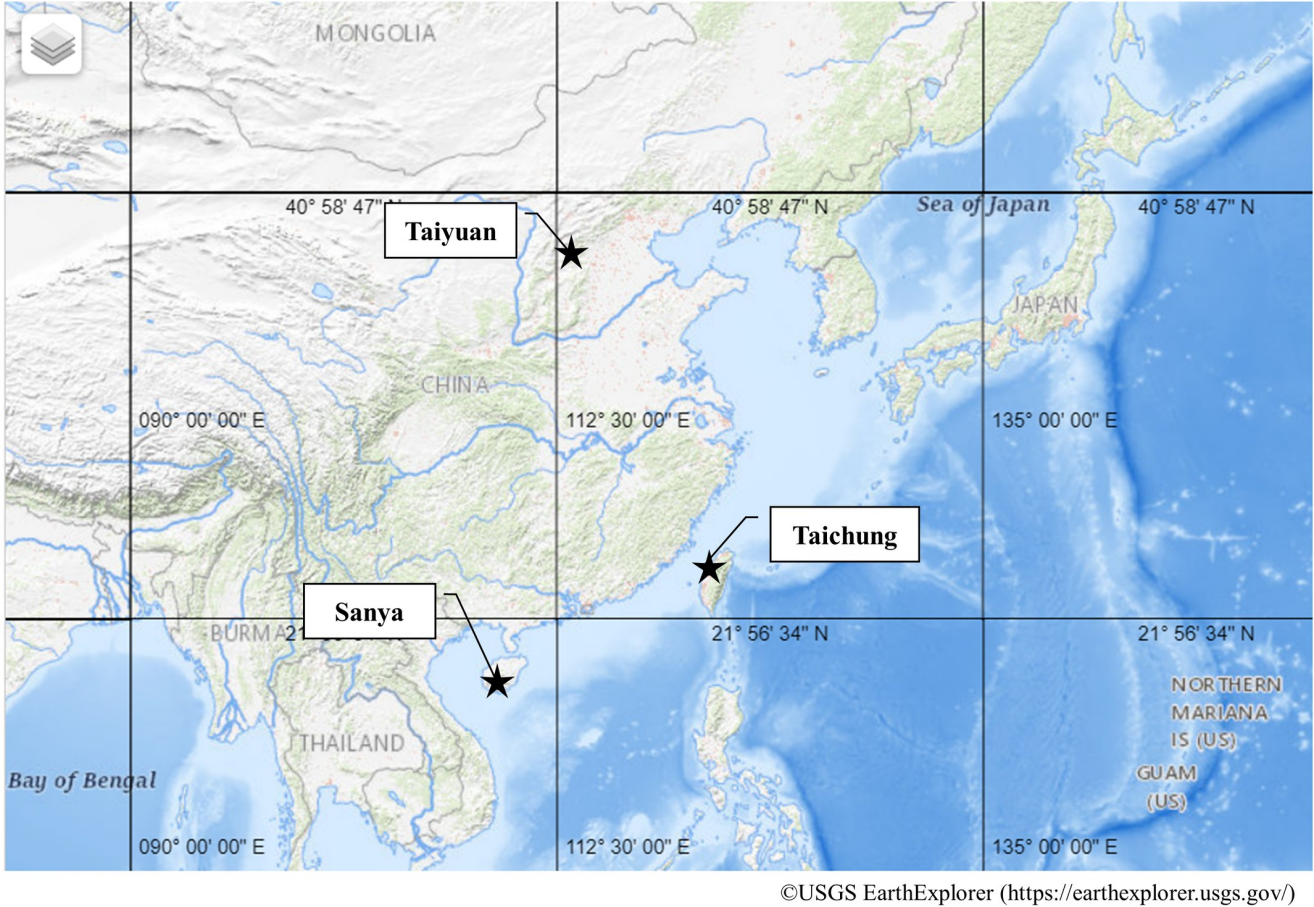
Locations of the three targeted regions. The map image resource is copyrighted by EarthExplorer of United States Geological Survey (USGS, https://earthexplorer.usgs.gov/).

Our previous work examined cataracts and UV exposure of residents in three regions of China and Taiwan with different UV intensities and annual temperatures (Fig 1, Table 1) [13]. From this study, we identified 1,547 subjects who had information on pterygium (presence or absence) in their right eye (444 in Sanya, 634 in Taiyuan, and 469 in Taichung). Because the pterygium prevalence of the right and left eyes did not significantly differ, the analysis was performed using the right eye (S1 Fig). The average age of participants was 60.4 ± 9.1 years (range 50–92). The axial length (AL) was measured using A-mode ultrasonography (NIDEK Inc., Fremont, CA) or the IOLMaster^TM^ (Carl Zeiss AG, Oberkochen, Germany). Participants completed a questionnaire with demographic characteristics, previous diagnosis of diabetes mellitus (DM), work history (indoor or outdoor workers), time spent outdoors, and the frequency of wearing prescription glasses, sunglasses, or hats while outdoors. This study adhered to the tenets of the Declaration of Helsinki and was performed with the approval (No. I376) of the Ethics Review Committee of Kanazawa Medical University. All subjects provided written informed consent.

**Table 1:** Climatic conditions in the three regions.

	Sanya	Taiyuan	Taichung
**UV intensity (J/m**^**2**^**)** ^¶^	233	142	213
**Annual average temperature (°C)**	26.9 ± 5.2	13.4 ± 9.0	24.3 ± 5.4

^¶^ NASA TOMS monthly erythemal UV data.

UV, ultraviolet.

### Methods

#### Classification of pterygium and cataract

Pterygium was graded based on the corneal progress rate as developed by Miyata et al. [45, 46] (S2 Fig). Three grades were diagnosed: grade 1 (mild, pterygium position up to one-third of the corneal diameter); grade 2 (moderate, up to two-thirds of the corneal diameter); and grade 3 (severe, more than two-thirds of the corneal diameter).

For eyes with pterygium, cataract was classified into three main types: COR, NUC, and PSC, and three subtypes: retrodots (RD), waterclefts (WC), and fiber folds (FF) [47, 48]. All cataracts were graded using slit-lamp images of the eyes by one ophthalmologist (HS) at maximum pupil dilation using the WHO cataract grading system [49]. COR opacities were also assessed by shape (axle-shaped, wedge-shaped, or ring-shaped), and the presence (CEN+) or absence (CEN-) of opacity in the central 3-mm diameter of the pupillary area; the RD and WC subtypes were determined by the Kanazawa Medical University Cataract Classification and Grading System as in our previous report [13], and FF was recorded as its presence or absence.

#### Prediction of cumulative ocular UV exposure (COUV) and UV intensity

The COUV was calculated as an estimated cumulative value using the below formulas [50] and area-specific UV intensities published by the U.S. National Aeronautics and Space Administration [51], adjusted for the use of glasses and hats [13]. The daily ocular UV exposure (OUV_day_) was determined by

$${OUV}_{day}={UV}_{day}\times (1-0.9\times {Gl}_{t})\times (1-0.2\times {Hat}_{t})$$

based on the following parameters: UV_day_, daily UV irradiance; protection factor of glasses, 0.9; Gl_t_ (use of glasses), always = 1, seldom = 0.5, no = 0; protection factor of hats, 0.2; and Hat_t_ (hat use), always = 1, seldom = 0.5, no = 0.

The mean daily ocular UV exposure (OUV_day_ ave) was calculated as

$${OUV}_{day}\;ave=[5\times {OUV}_{day}(weekdays)+2\times {OUV}_{day}(weekend\;days)]÷7$$

whereas the cumulative ocular UV exposure (COUV) was determined using the formula

$$COUV=\sum_{y=20}^{Age}\left[Ly\sum_{d=0}^{365}\left({OUV}_{day}\;ave\right)\right]$$

with COUV being the estimated cumulative ocular UV exposure and Ly the location factor.

The prevalence of pterygium and the correlations with COUV were considered after dividing the COUV values into five levels: < 5, 5 to < 10, 10 to < 15, 15 to < 20, and ≥ 20 (×10^6^).

#### Statistical analysis

All statistical analyses were performed using SPSS Statistics version 24 (IBM Japan, Ltd., Tokyo, Japan). The Kruskal-Wallis test for comparison among three unpaired groups was used to assess differences in the average COUV between the regions, whereas Welch’s t-test for the mean of two groups was used to test the difference in COUV for eyes with/without pterygium. Logistic regression analysis was used to calculate the odds ratio (OR) and 95% confidence intervals (CI) for risk factors for pterygium, cataract risk in eyes with pterygium, and cataract risk with increasing pterygium grade, controlling for the subject’s age, sex, work history, AL, and DM. Multiple regression analysis was also used to examine the associations between pterygium and some other factors. A p value of < 0.05 was considered statistically significant.

## Results

### Characteristics of subjects with pterygium

Pterygium was found in 58.8% (261/444, 95% CI for overall prevalence: 54.6–63.8) of the subjects in Sanya, 11.8% (75/634, 95% CI: 9.3–14.3) of participants in Taiyuan, and 5.3% (25/469, 95% CI: 3.3–7.4) of subjects in Taichung, with an overall prevalence of 23.3% (361/1,547, 95% CI: 21.2–25.4) (Table 2). The prevalence of pterygium increased with age in all three regions, reaching 82.8% in Sanya for subjects over 80 years old.

**Table 2:** Prevalence of pterygium.

		Sanya	Taiyuan	Taichung	Overall
**Subjects (n)**		444	634	469	1,547
**Age (y)**	**Mean ± SD**	60.2 ± 10.4	60.4 ± 8.9	60.5 ± 8.0	60.4 ± 9.1
	**50–59**	129 / 249 (51.8%)	29 / 336 (8.6%)	9 / 258 (3.5%)	167 / 843 (19.8%)
	**60–69**	60 / 95 (63.2%)	19 / 163 (11.7%)	9 / 129 (7.0%)	88 / 387 (22.7%)
	**70–79**	48 / 71 (67.6%)	25 / 119 (21.0%)	6 / 74 (8.1%)	79 / 264 (29.9%)
	**≥ 80**	24 / 29 (82.8%)	2 / 16 (12.5%)	1 / 8 (12.5%)	27 / 53 (50.9%)
	**Total**	261 / 444 (58.8%)	75 / 634 (11.8%)	25 / 469 (5.3%)	361 / 1,547 (23.3%)
**Sex**	**Male**	86 / 164 (52.4%)	42 / 240 (17.5%)	20 / 193 (10.4%)	148 / 597 (24.8%)
	**Female**	175 / 280 (62.5%)	33 / 394 (8.4%)	5 / 276 (1.8%)	213 / 950 (22.4%)

n, number; y, years; SD, standard deviation.

Of the 361 eyes with pterygium, outdoor workers accounted for 86.1% (Table 3). There were fewer outdoor workers in Taichung, an industrial city, than in Sanya and Taiyuan which are rural areas with many farmers. DM was observed only in 2.5% of the subjects with pterygium.

**Table 3:** Ocular factors associated with pterygium.

	Sanya	Taiyuan	Taichung	Overall
**Subjects (n) with pterygium**	261	75	25	361
**Outdoor workers**	245 (93.9%)	61 (81.3%)	5 (20.0%)	311 (86.1%)
**AL (Mean ± SD)**	23.2 ± 1.0	22.7 ± 0.9	24.1 ± 2.0	23.2 ± 1.1
**DM**	2 (0.8%)	3 (4.0%)	4 (16.0%)	9 (2.5%)

n, number; AL, axial length; SD, standard deviation; DM, diabetes mellitus.

The adjusted risk of pterygium increased significantly with age (OR: 1.029 [95% CI: 1.016−1.043], p < 0.001), but was not significantly associated with sex (Table 4). Outdoor workers were approximately five times more likely to have pterygium (OR: 5.257 [95% CI: 3.730–7.411], p < 0.001) than indoor workers. The risk of pterygium increased nonsignificantly with increasing AL but was significantly lower when controlled for DM (p = 0.001).

**Table 4:** Odds ratio by factors for risk of pterygium.

	Odds ratio (95% CI)	p value
**Age**	1.029 (1.016 − 1.043)	< 0.001**
**Sex (male)**	1.100 (0.845 − 1.431)	0.480
**Outdoor workers**	5.257 (3.730 − 7.411)	< 0.001**
**AL**	1.086 (0.975 − 1.211)	0.135
**DM**	0.313 (0.154 − 0.638)	0.001*

Logistic regression analysis adjusted for age, sex, work history, AL, and DM

* p < 0.01

** p < 0.001. 95% CI, 95% confidence interval for overall prevalence; AL, axial length; DM, diabetes mellitus.

### Relationship between the prevalence of pterygium and COUV

**Fig 2:**
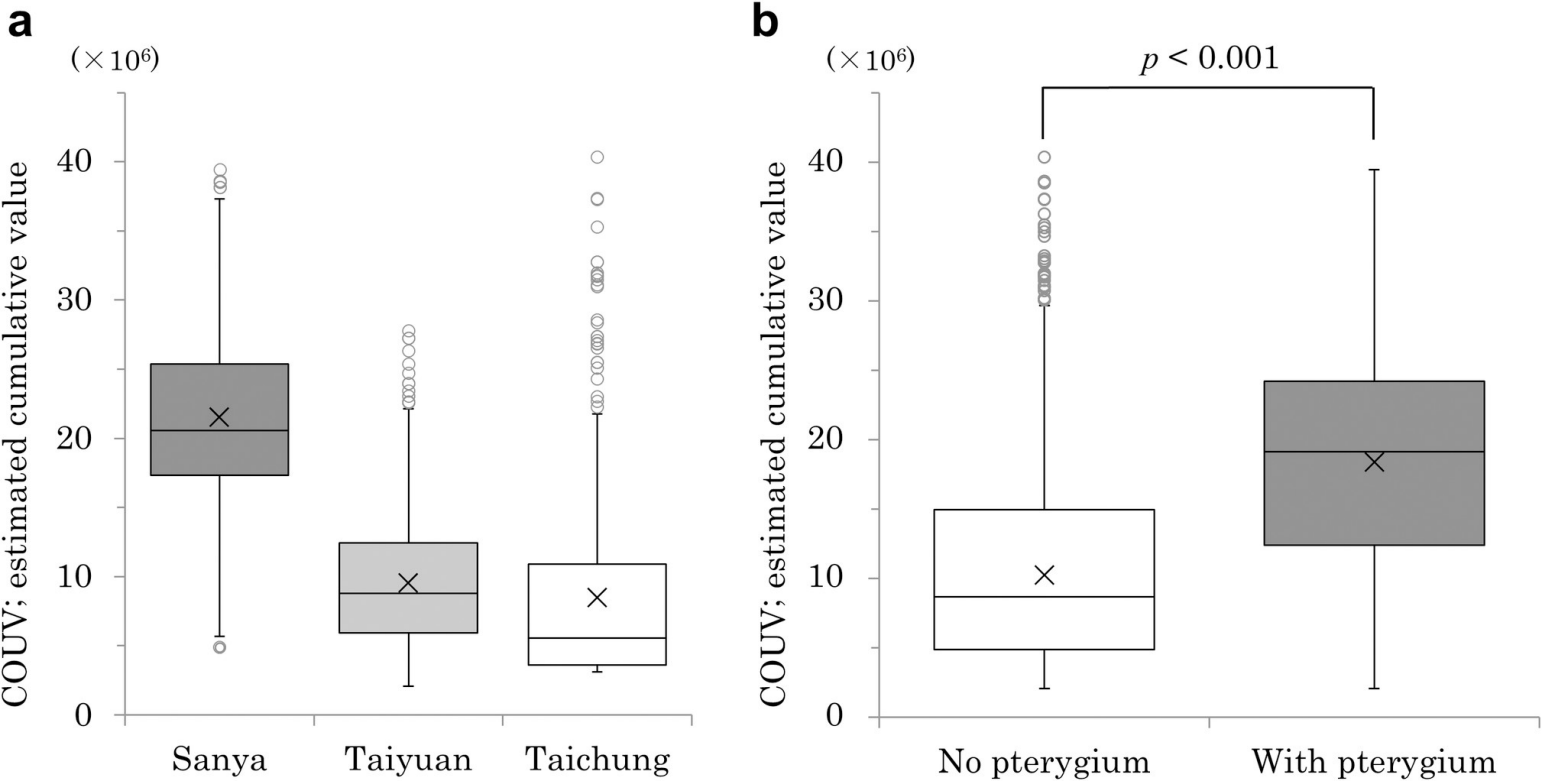
Relationship between pterygium and COUV. a: The average COUV was not significantly different among the three regions. b: The average COUV of eyes with pterygium was 1.86-fold higher than for eyes without pterygium. ×: average value, ○: outlier. COUV, cumulative ocular UV exposure.

As ocular UV exposure dose is greatly affected by the individual’s time spent outdoors and usage of UV eye protection equipment, we focused on the COUV of individuals. The average COUV values in each region were 21.5 ± 6.7 (×10^6^) in Sanya, 9.5 ± 5.1 (×10^6^) in Taiyuan, and 7.4 ± 6.3 (×10^6^) in Taichung; these values were not significantly different (Fig 2A). However, COUV was 1.86-fold higher for the 361 eyes with pterygium than those without (18.4 ± 8.5 [×10^6^] vs. 9.9 ± 7.3 [×10^6^], respectively; p < 0.001) (Fig 2B).

**Fig 3:**
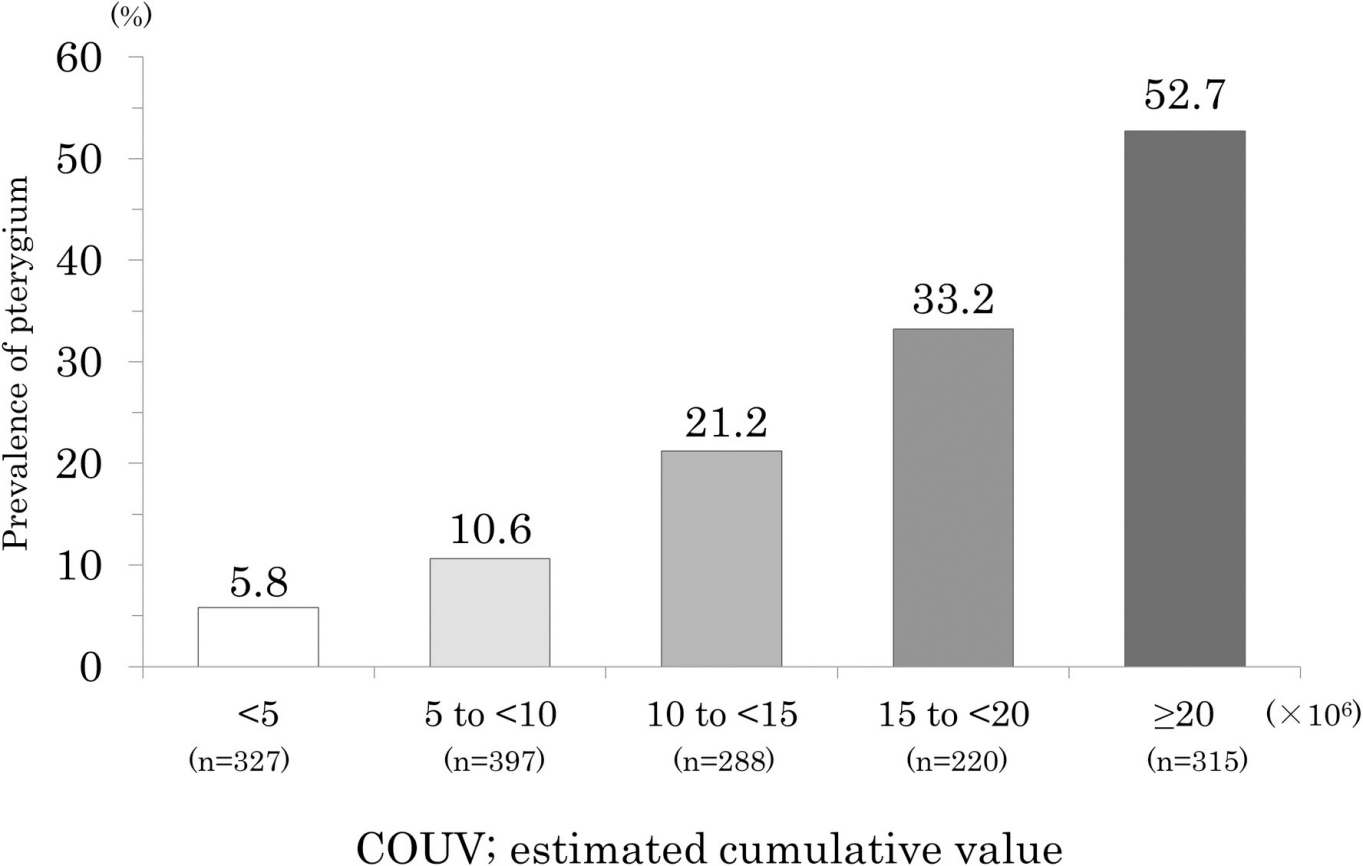
Relationship between pterygium and COUV levels. The COUV values were divided into five levels: < 5, 5 to < 10, 10 to < 15, 15 to < 20, and ≥ 20 (×10^6^), and the relationship between pterygium and COUV was investigated. COUV, cumulative ocular UV exposure.

The mean COUV value for all subjects was 11.7 (×10^6^). To verify the correlation between COUV and pterygium, COUV of all subjects was divided into five levels of 5 (×10^6^) each: < 5, 5 to < 10, 10 to < 15, 15 to < 20, and ≥ 20 (×10^6^). Logistic regression analysis showed that the risk of pterygium increased significantly with increasing COUV level (OR: 2.043 [95% CI: 1.844–2.257], p < 0.001) (Fig 3).

Table 5 presents the association between pterygium and factors like age, sex, work history, AL, DM, and COUV via multiple regression analysis, showing significant relationships among age, outdoor workers, DM, and COUV. In addition, the parameter COUV had a higher coefficient (β) value than being an outdoor worker, indicating a stronger association of the COUV with the presence of a pterygium. A significant negative tendency was detected in the association between DM and pterygium.

**Table 5:** Multiple regression analysis on pterygium.

	Coefficient (β)	SE	95% CI	p value
**Age**	0.051	0.001	0.000 − 0.005	0.032*
**Sex (male)**	0.010	0.021	-0.032 − 0.050	0.676
**Outdoor workers**	0.055	0.012	0.004 − 0.053	0.020*
**AL**	0.017	0.008	-0.010 − 0.022	0.471
**DM**	-0.064	0.037	-0.174 − -0.028	0.007**
**COUV**	0.378	0.000	0.098 − 0.126	< 0.001***

Multiple regression analysis adjusted for age, sex, work history, AL, DM, and COUV

* p < 0.05

** p < 0.01

*** p < 0.001.

SE, spherical equivalent; 95% CI, 95% confidence interval for overall prevalence; AL, axial length; DM, diabetes mellitus; COUV, cumulative ocular UV exposure.

### Risk of cataract in eyes with pterygium

Table 6 presents the prevalence of cataract, including comorbid opacities, in the 361 eyes with pterygium, as well as the cataract risk in eyes with pterygium compared with those without pterygium. A total of 179 eyes (48.5%) were diagnosed with cataract; NUC and PSC opacities were found in 41.6% (OR: 6.557 [95% CI: 4.684–9.179], p < 0.001) and 11.1% (OR: 1.976 [95% CI: 1.248–3.131], p = 0.004) of the eyes, respectively. Contrastingly, COR was not significantly associated with pterygium (26.0%, OR: 1.119 [95% CI: 0.818–1.531], p = 0.483), although it has been reported to be associated with ocular UV exposure [4, 52, 53]. After dividing COR opacity into five detailed types, increased risk was detected in eyes with axle-shaped opacity (CEN-) (OR: 2.336 [95% CI: 1.266–4.309], p = 0.007) and ring-shaped opacity (OR: 2.117 [95% CI: 1.189–3.771], p = 0.011). Regarding cataract subtypes, RD was associated with a significant increase in the risk of pterygium (OR: 2.553 [95% CI: 1.908–3.416], p < 0.001), whereas WC showed a significant decrease in the risk (OR: 0.542 [95% CI: 0.338–0.869], p = 0.011). From the point of view of the three primary types of cataract, the number of eyes with only NUC, COR, or PSC was 68 (18.8%), 25 (6.9%), and 2 (0.5%), respectively.

**Table 6:** Cataract prevalence and risk in eyes with pterygium.

			Prevalence (95% CI)	Odds ratio^¶^ (95% CI)	p value
**COR**			26.0% (21.5–30.6)	1.119 (0.818–1.531)	0.483
	**Axle-shaped opacity**	**CEN (-)**	7.2% (4.5–9.9)	2.336 (1.266 − 4.309)	0.007**
		**CEN (+)**	5.5% (3.2–7.9)	1.135 (0.532 − 2.421)	0.743
	**Wedge-shaped opacity**	**CEN (-)**	11.4% (8.1–14.6)	0.648 (0.407 − 1.032)	0.067
		**CEN (+)**	8.6% (5.7–11.5)	0.747 (0.397 − 1.406)	0.367
	**Ring-shaped opacity**		6.9% (4.3–9.5)	2.117 (1.189 − 3.771)	0.011*
**NUC**			41.6% (36.5–46.6)	6.557 (4.684 − 9.179)	< 0.001***
**PSC**			11.1% (7.8–14.3)	1.976 (1.248 − 3.131)	0.004**
**RD**			44.0% (38.9–49.2)	2.553 (1.908 − 3.416)	< 0.001***
**WC**			7.2% (4.5–9.9)	0.542 (0.338 − 0.869)	0.011*
**FF**			15.8% (12.0–19.6)	1.033 (0.708 − 1.505)	0.868

^¶^ Odds ratio for cataract risk in eyes with pterygium compared with those without pterygium, obtained by logistic regression analysis adjusted for age, sex, axial length, and diabetes mellitus

* p < 0.05

** p < 0.01

*** p < 0.001.

95% CI, 95% confidence interval for overall prevalence; COR, cortical; CEN, opacity in the 3-mm central pupillary area; NUC, nuclear; PSC, posterior subcapsular cataract; RD, retrodots; WC, waterclefts; FF, fiber folds.

### Pterygium grade and cataract risk

For the 361 eyes with pterygium, the cataract risk for each additional grade of pterygium is shown in Table 7. After examining the four types of cataracts (COR axle-shaped opacity with CEN (-) and ring-shaped opacity, NUC, PSC, and RD), whose risks increased significantly in eyes with pterygium as shown in Table 6, no significant correlation for increasing pterygium grade was found in any type of cataract, revealing that the cataract risk was not increased with pterygium progression. In addition, there was no relationship between pterygium grade and COUV.

**Table 7:** Cataract prevalence and risk with increasing pterygium grade.

	Prevalence per pterygium grade (95% CI)			Odds ratio^¶^ (95% CI)	p value
	1 (n = 247)	2 (n = 76)	3 (n = 38)		
**COR Axle-shaped opacity CEN (-)**	5.7% (2.8–8.6)	9.2% (3.8–18.1)	13.2% (4.4–28.1)	1.733 (0.959–3.131)	0.069
**COR Ring-shaped opacity**	6.5% (3.4–9.5)	6.6% (2.2–14.7)	10.5% (2.9–24.8)	1.249 (0.685–2.275)	0.468
**NUC**	40.1% (34.0–46.2)	44.7% (33.6–55.9)	44.7% (28.9–60.5)	1.096 (0.750–1.601)	0.637
**PSC**	12.1% (8.1–16.2)	7.9% (3.0–16.4)	10.5% (2.9–24.8)	0.728 (0.401–1.320)	0.296
**RD**	42.9% (36.7–49.1)	47.4% (36.1–58.6)	44.7% (28.9–60.5)	1.095 (0.775–1.549)	0.606

^¶^ Odds ratio for cataract risk in eyes with pterygium for each additional grade of pterygium, obtained by logistic regression analysis adjusted for age, sex, axial length, and diabetes mellitus.

95% CI, 95% confidence interval for overall prevalence; COR, cortical cataract; CEN, opacity in the 3-mm central pupillary area; NUC, nuclear cataract; PSC, posterior subcapsular cataract; RD, retrodots.

## Discussion

In this study, we found a significant statistical relationship between ocular UV exposure and risk of pterygium development in people from three central Asian regions with different UV intensities. After adjusting for age, sex, work history, axial length, and diabetes mellitus, NUC was the cataract type most likely to be associated with pterygium, followed by RD, COR with axle-shaped opacity (CEN-), COR with ring-shaped opacity, and PSC.

Many epidemiological surveys have been conducted on the prevalence and risk factors of pterygium. In this study, we focused on the Han people living in the three regions in the “pterygium zone” within the latitudes 37° north and 37° south of the equator [40, 54]. The largest number of pterygium-affected eyes were in Sanya, which also had the highest UV intensity of the three regions. On the other hand, the lowest prevalence of pterygium was in Taichung, whereas Taiyuan had the lowest UV intensity. This suggests that UV intensity does not have a strictly linear relationship with the prevalence of pterygium. Both the prevalence and adjusted risk of pterygium increased with age in all three regions.

After controlling for time spent outdoors, age, and the use of sunglasses, eyeglasses, and hats, the average COUV was significantly higher for subjects with pterygium, and the risk of pterygium increased significantly with increasing COUV; thus, these results indicate a clear association between COUV and pterygium. On the other hand, pterygium was observed in eyes with low COUV levels (less than 5×10^6^) as well. In a previous report, Australian residents who had spent the first 5 years of their lives in areas at latitudes less than 30° had an approximately 40-fold higher risk of pterygium than those at latitudes greater than 40°, and the risk of pterygium increased approximately 20-fold for those with the longest time spent outdoors in those early years [29]. A survey of over 100,000 aboriginal and non-aboriginal Australians in rural areas reported that their pterygium prevalence of 3.41% and 1.08%, respectively, were correlated with UV radiation and that lifestyle differences may cause the observed racial and sex differences [6]. However, the study did not assess UV exposure in individuals less than 20 years old, distinguish between chronic and acute high-level UV exposure history, or record individual differences in UV sensitivity. These issues merit further study.

Wang et al. reported that the overall prevalence of pterygium in 1,910 Han and 741 Mongolian people was 6.4% and that pterygium was significantly correlated with age, outdoor occupation, and time spent in rural areas [43]. In the Brazilian Amazon, a region with high UV exposure, randomly selected adults had a 58.8% prevalence of pterygium; the prevalence was higher for the male sex, older age, lower education, and rural residence, and it was lower for higher education [55]. The prevalence of pterygium in that study was similar to that of Sanya (58.8%) in our study. Since there are many occupational farmers in Sanya and Taiyuan, the increased amount of outdoor activity time may have contributed to the high prevalence and increased risk. In our study, outdoor workers had a 5.26-fold higher risk of pterygium compared to participants working mainly indoors. COUV may thus be a more important factor than occupational exposure.

In this study, we investigated three main types and three subtypes of cataract, additionally dividing COR into five opacity types. We found NUC to be the most frequent type of cataract, and to have the highest associated risk for the development of pterygium. While COR-type cataracts were the second most prevalent type, the two subtypes of axle-shaped opacity (CEN-) and ring-shaped opacity were significantly associated with pterygium. This result is equivalent to the finding by Modenese et al. [21] demonstrating that not only cortical cataract is associated with cumulative solar UV exposure, but also the nuclear forms which were in our studies the most frequent types in subjects with pterygium, most of them outdoor workers with high COUV levels. RD are opacities from calcium phosphate deposits in the surface of the lens nucleus whereas WC result from water retention around the lenticular Y-shaped suture. However, the mechanisms involved in their formation have not been clarified to date [56, 57]. Recent studies have reported that lenses with WC have low levels of Cx50 protein involved in cell-cell gap junctions, which may be degraded by autophagy [58]. The pterygium risk increase with RD and its decrease with WC suggest an effect of decreased lenticular moisture. This is difficult to test, however, as currently, there are no practical methods of measuring lenticular moisture in the living human body.

This study showed that pterygium grade was not significantly associated with cataract risk and COUV. Factors other than UV exposure can be considered for the progression of pterygium; however, it is difficult to investigate these factors in this type of cross-sectional survey. Regarding the progression of pterygium, it is necessary to study this aspect using a longitudinal survey for each subject, which should be considered in the future. In this study, it was revealed that ocular UV exposure was associated only with the incidence of pterygium.

There are very few reports on the relationship between pterygium and cataract. A recent study in two rural areas of China reported that the risk factors for pterygium included latitude, age-related cataract, sex (mostly female), and age [59]. In a clinical study at an emergency county hospital in Romania, 23 of 84 patients with pterygium also had cataracts [40]. In an epidemiological study in Ecuador, it was indicated that both pterygium and aging cataract may result from cumulative UV exposure [60]. Although these reports examined the existence of pterygium and cataract, none provided details of cataract types. Thus, we believe that this is the first report on the association between pterygium and cataract subtypes.

The study has several limitations. First, we only investigated the right eyes of the subjects for the possibility that pterygium onset could be an objective indicator of high UV exposure and the relationship between pterygium and cataract from ocular UV exposure. There may be crosswise differences in eyes with pterygium due to occupational factors [61, 62], such as in long-distance drivers, whose eyes on the window side may have pterygium. However, the subjects in this study were mainly farmers, with nearly the same pterygium grade in both right and left eyes. In this epidemiological study, only the right eye was investigated to objectively assess it without bias and to examine any relationship of pterygium with cataract. Second, COUV, an estimation of ocular UV exposure dosage, was calculated from answers to questionnaires on the time spent outdoors and the use of eyeglasses/sunglasses and hats. Records of individuals’ past activities depend on their memory and the standard of outdoor activity varies among individuals, causing variability in answers, lower reliability, and possible recall bias. Furthermore, the COUV calculation includes a 90% cut (factor 0.9) for all eyeglasses/sunglasses and 20% cut (factor 0.2) for hats. However, accurate cut rates may differ depending on the type and shape of the eyeglasses/sunglasses and hats. COUV is also a prediction formula and has limitations in evaluating actual UV exposure dose. We found that the presence of both pterygium and COUV is a useful index to evaluate ocular UV exposure dose, and we will continue to use them as evaluation tools in future research. Third, many factors, including race, education, long AL (high myopia), DM, smoking, steroid use, and UV and ionizing radiation exposure, have been reported as risk factors for cataract development. All subjects in our study were of the Han ethnic group and were analyzed after adjusting for AL and DM; future studies should strive for greater diversity in racial backgrounds and environmental factors (such as education and work history). In addition, as DM was detected only from the questionnaires without measuring blood glucose levels through blood tests, the reliability may be somewhat low; consequently, it is likely that the risk of pterygium in DM subjects was low in this study. Studies have reported that fibroblast cells derived from patients with noninsulin-dependent DM (type II) have decreased cell proliferation ability [63], which suggests a relationship between blood glucose concentration and cell proliferation ability and indicates that patients with DM may have less frequently pterygium composed of fibroblast cells. Although a previous report showed that DM is not a risk factor for pterygium [64], we have not found any literature reporting that DM decreases the risk of pterygium.

## Conclusions

Our study found COUV to be a significant predictor of the risk of pterygium development. Thus, pterygium may be a useful indicator of ocular UV exposure in other eye diseases. Four cataract types (COR with axle-shaped opacity without opacity in the central pupillary area and ring-shaped opacity, NUC, PSC, and RD) were associated with pterygium. These results support the existence of a relationship between ocular UV exposure and specific cataract type.

## Supporting information

S1 Fig Prevalence of pterygium or each opacity type of cataracts in the right and left eyes. The prevalence between right and left eyes were not significantly different (chi-square test). COR, cortical cataract; CEN-/+, absence or presence of the central opacity in the pupillary area; NUC, nuclear cataract; PSC, posterior subcapsular cataract; RD, retrodots; WC, waterclefts; FF, fiber folds.
(TIF)

S2 Fig Pterygium Grading System based on the corneal progress rate. Grade 1: mild, pterygium position up to one-third of the corneal diameter, Grade 2: moderate, pterygium position up to two-thirds of the corneal diameter, Grade 3: severe, pterygium position more than two-thirds of the corneal diameter.
(TIF)

S1 Table Demographics of the cross-sectional samples examined for cataract prevalence and risk in eyes with pterygium [Table 6]. Some cases overlap in cataract type classification.
(TIF)

S2 Table Demographics of the cross-sectional samples examined for cataract prevalence and risk with increasing pterygium grade [Table 7]. Some cases overlap in cataract type classification.
(TIF)
