# Supplementary figures and images for: Association among pterygium, cataracts, and cumulative ocular ultraviolet exposure: A cross-sectional study in Han people in China and Taiwan
Source: PLoS One. 2021 Jun 15;16(6):e0253093. doi: 10.1371/journal.pone.0253093 (PMC8205177; doi:10.1371/journal.pone.0253093)

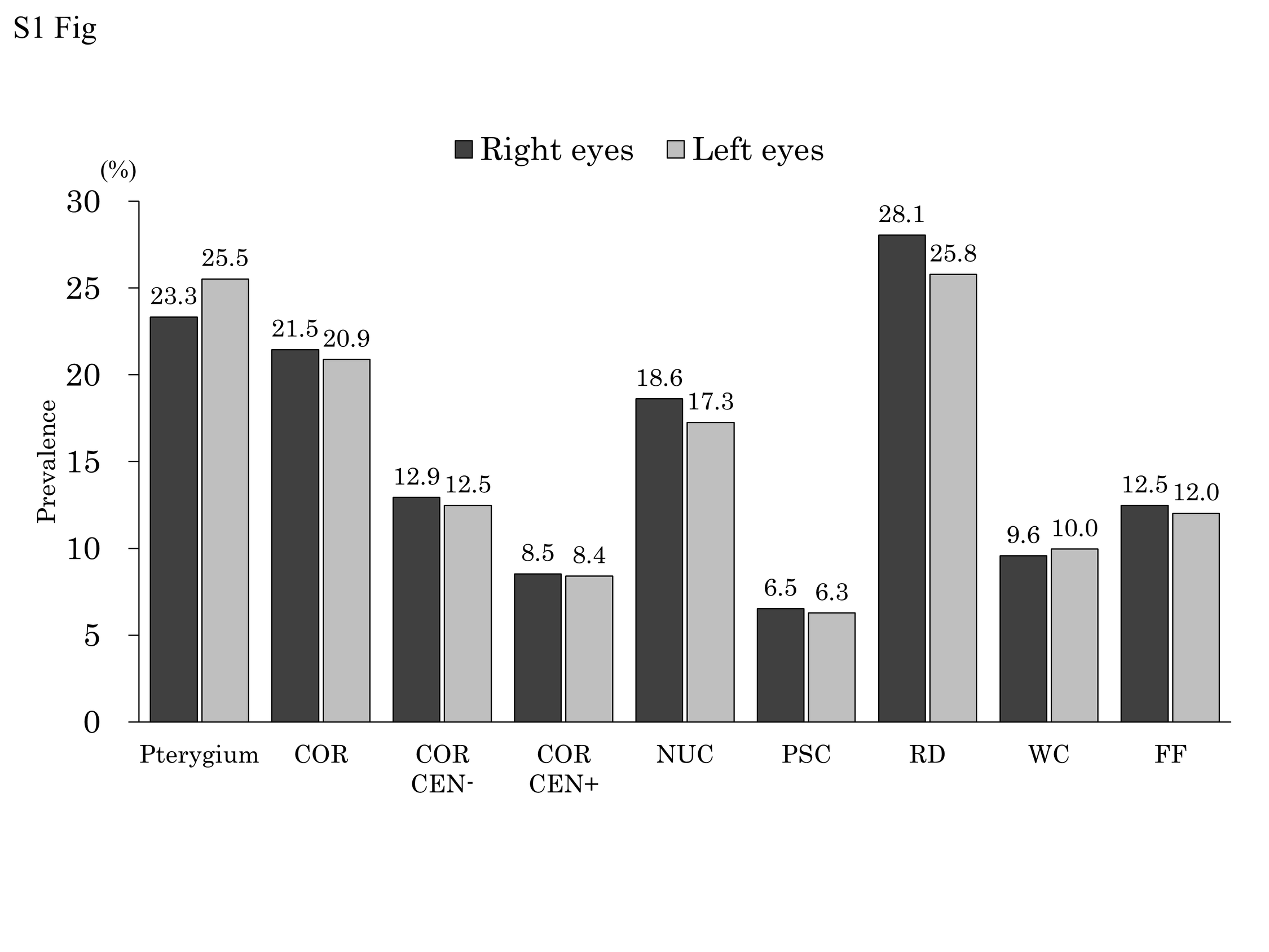

Supplement: S1 Fig — The prevalence between right and left eyes were not significantly different (chi-square test). COR, cortical cataract; CEN-/+, absence or presence of the central opacity in the pupillary area; NUC, nuclear cataract; PSC, posterior subcapsular cataract; RD, retrodots; WC, waterclefts; FF, fiber folds. (TIF) [file pone.0253093.s001.tif]

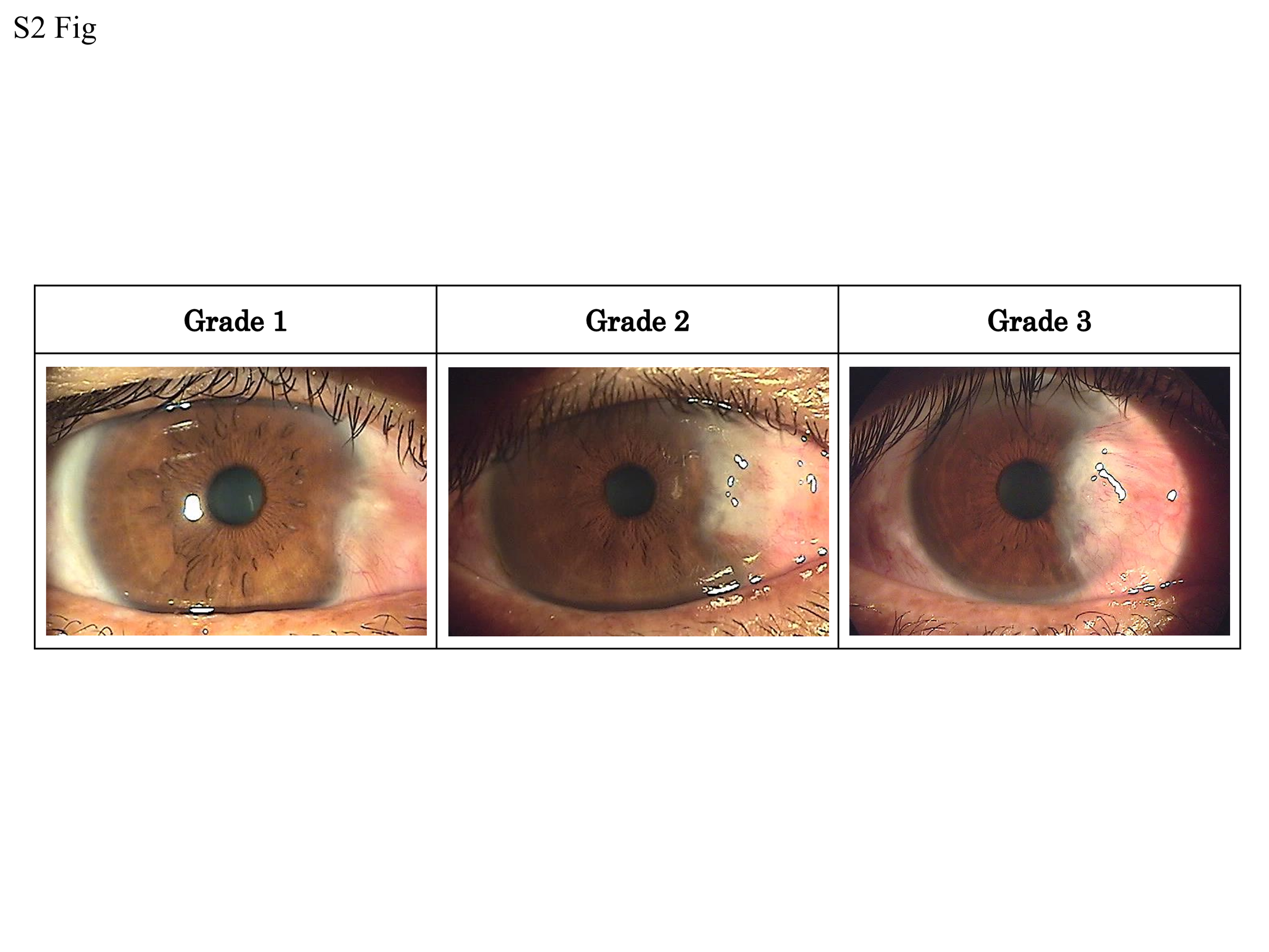

Supplement: S2 Fig — Grade 1: mild, pterygium position up to one-third of the corneal diameter, Grade 2: moderate, pterygium position up to two-thirds of the corneal diameter, Grade 3: severe, pterygium position more than two-thirds of the corneal diameter. (TIF) [file pone.0253093.s002.tif]

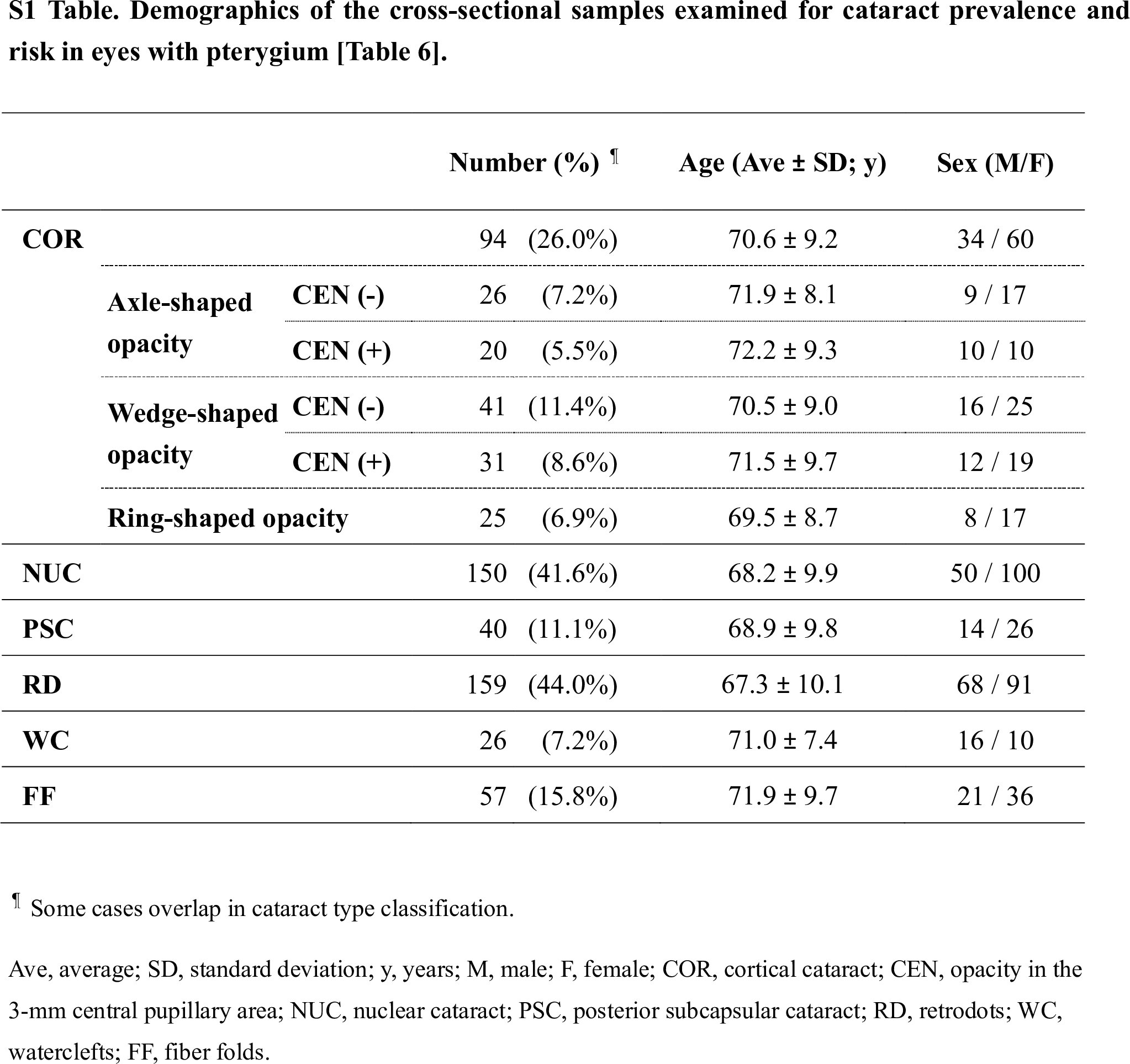

Supplement: S1 Table — Some cases overlap in cataract type classification. (TIF) [file pone.0253093.s003.tif]

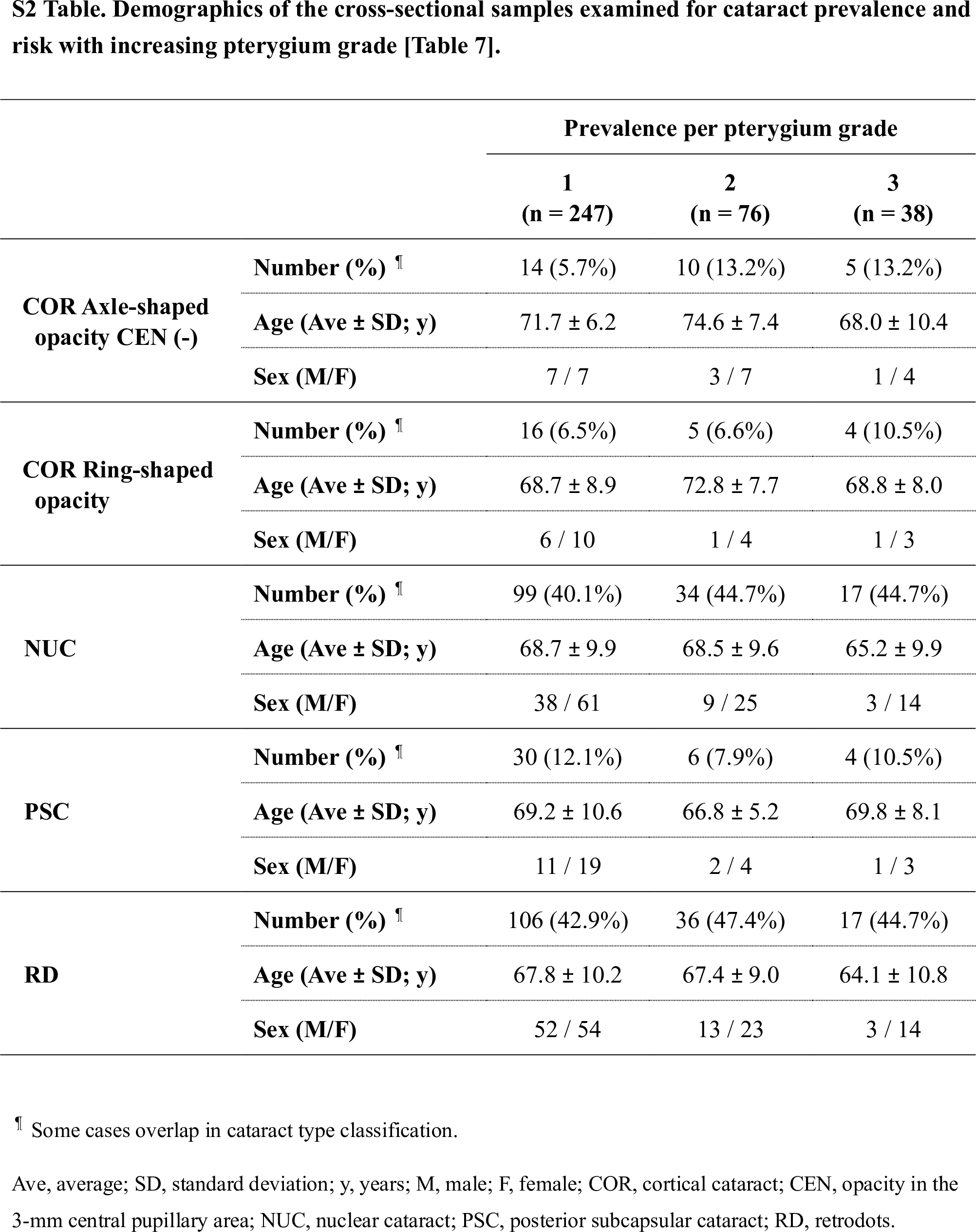

Supplement: S2 Table — Some cases overlap in cataract type classification. (TIF) [file pone.0253093.s004.tif]
